# Therapeutic strategies and genetic profile comparisons in small cell carcinoma and large cell neuroendocrine carcinoma of the lung using next-generation sequencing

**DOI:** 10.18632/oncotarget.22426

**Published:** 2017-11-14

**Authors:** Masaoki Ito, Yoshihiro Miyata, Shoko Hirano, Shingo Kimura, Fumiko Irisuna, Kyoko Ikeda, Kei Kushitani, Yasuhiro Tsutani, Daisuke Ueda, Norifumi Tsubokawa, Yukio Takeshima, Morihito Okada

**Affiliations:** ^1^ Department of Surgical Oncology, Research Institute for Radiation Biology and Medicine, Hiroshima University, Hiroshima, Japan; ^2^ Analysis Center of Life Science, Natural Science Center for Basic Research and Development, Hiroshima University, Hiroshima, Japan; ^3^ Department of Pathology, Graduate School of Biomedical & Health Sciences, Hiroshima University, Hiroshima, Japan

**Keywords:** next generation sequencing, small cell lung cancer, large cell neuroendocrine carcinoma, genetic variants, therapeutic targets

## Abstract

Small cell lung cancer (SCLC) and large cell neuroendocrine carcinoma (LCNEC) of the lung are classified as variants of endocrine carcinoma and subdivided into pure or combined type. Clinical benefit of target therapy has not been established in these tumors. This study aimed to compare genetic and clinicopathological features between SCLC and LCNEC or pure and combined types, and explore the possibility of target therapy using next-generation sequencing. In 13 SCLC and 22 LCNEC cases, 72 point mutations, 19 deletions, and 3 insertions were detected. As therapeutically targetable variants, mutations in *EGFR* (L858R), *KRAS* (G12D, G12A, G12V), and *PIK3CA* (E545K) were detected in 5 cases. The case harboring EGFR mutation showed response to EGFR-tyrosine kinase inhibitor. However, there are no clinicopathological features associated with therapeutically targetable cases. And there was no significant genetic feature between SCLC and LCNEC or pure and combined types. In conclusion, although patients with SCLC and LCNEC may benefit from target therapy, they were not identifiable by clinicopathologic background. And there was not significant genetic difference between SCLC and LCNEC, including between pure and combined types. Classifying SCLC and LCNEC in same category is reasonable. However, distinguishing the pure type from combined type was not validated. Comprehensive genetic analysis should be performed to detect targetable variants in any type of SCLC and LCNEC.

## INTRODUCTION

Small cell lung cancer (SCLC) and large cell neuroendocrine carcinoma (LCNEC) of the lung are highly malignant phenotypes of lung cancer. They are morphologically distinguishable, but are classified as variants of endocrine carcinoma in the 2015 World Health Organization classification [1]. The optimal treatments for SCLC and LCNEC are similar. Chemotherapy with conventional cytotoxic agents is recommended for SCLC [2, 3] and has been also used for LCNEC [4, 5]. This strategy has not changed for at least a decade. Although SCLC and LCNEC are often subdivided into pure type or combined type, the clinical benefit of this subclassification is also unclear.

Recently, next-generation sequencing (NGS) has revealed the genetic profiles of some types of lung cancers, and it has the benefit of detecting therapeutic targets simultaneously. The aim of this study was to detect therapeutic targets, reveal specific characteristics of cases with targetable genetic variants, and compare the genetic and clinicopathological features between SCLC and LCNEC or between pure type and combined type using NGS.

## RESULTS

### Clinicopathological features, NGS results, and validation

Forty SCLC and LCNEC cases with available frozen sections were reviewed. A library was not established in 3 cases and NGS did not provide readable data in 2 cases; thus, the genetic profiles of 35 cases were assessed using NGS. Characteristics of the 35 included patients are shown in Table 1. Histologically, the cases were divided into pure SCLC (N = 2), combined SCLC (N = 11), pure LCNEC (N = 8), and combined LCNEC (N = 14). The median patient age was 70 years. Thirty patients (85.7%) were male and 31 (88.6%) were current or ex-smokers.

**Table 1:** Clinicopathological characteristics of the enrolled small cell lung cancer (SCLC) and large cell neuroendocrine carcinoma (LCNEC) cases (N = 35)

Clinicopathological characteristic	Number (%)
Age, years	
Median	70
Range	47–84
Sex	
Male	30 (85.7)
Female	5 (14.3)
Smoking status	
Smoker	31 (88.6)
Never-smoker	4 (11.4)
Histological subtype	
Pure SCLC	2 (5.7)
Combined SCLC	11 (31.4)
Pure LCNEC	8 (22.9)
Combined LCNEC	14 (40.0)
Accompanied component in combined SCLC	Number (% in all combined SCLC)
LCNEC	9 (81.8)
Adenocarcinoma	4 (36.4)
Squamous cell carcinoma	1 (9.1)
Large cell carcinoma	2 (18.2)
Accompanied component in combined LCNEC	Number (% in all combined LCNEC)
Adenocarcinoma	10 (71.4)
Squamous cell carcinoma	6 (42.9)
Large cell carcinoma	0 (0)

**Figure 1:**
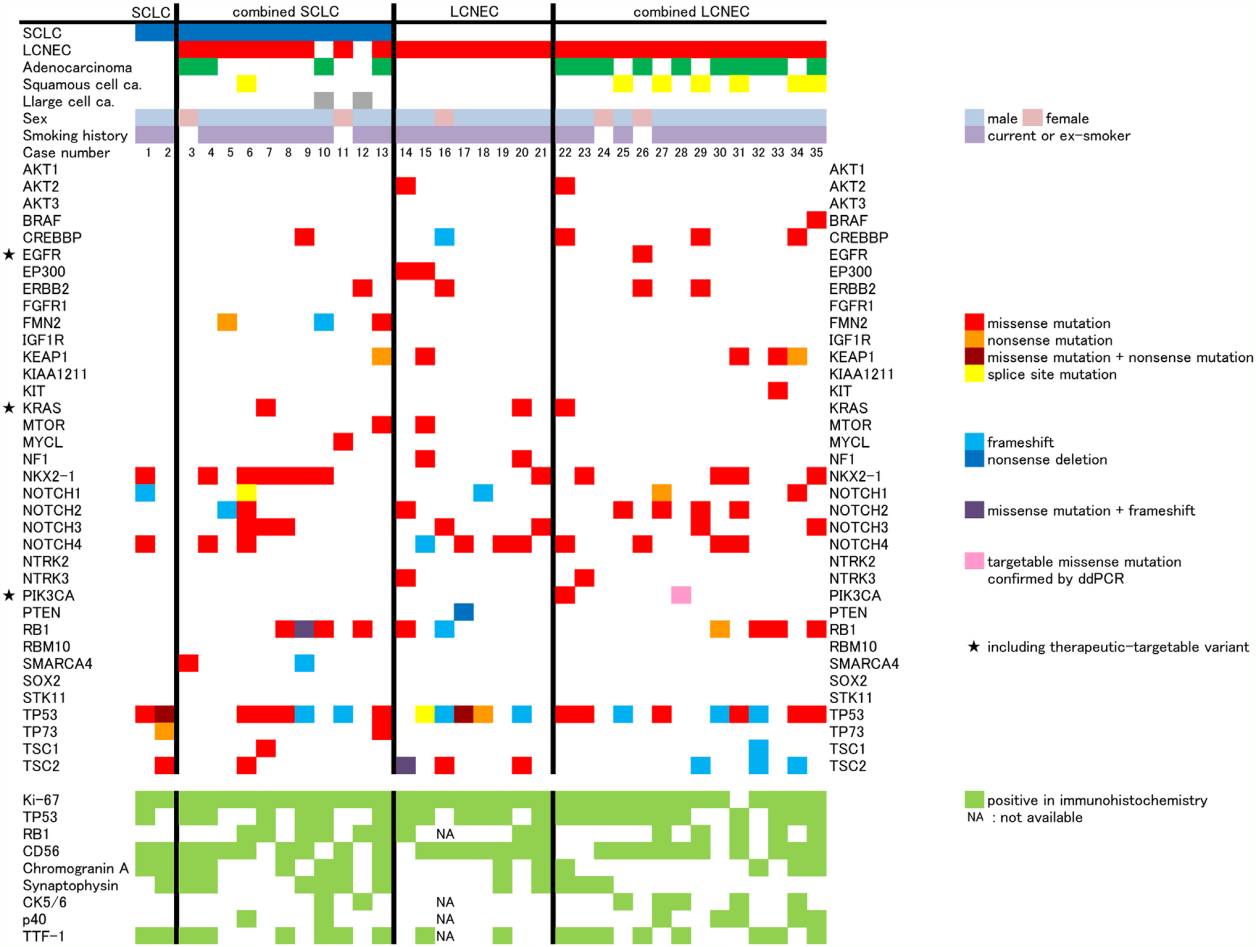
Result of next-generation sequencing and immunohistochemistry analysis Histological subtype is on the horizontal axis. On the vertical axis, accompanied component, sex, smoking history, case number, examined gene by next generation sequencing, and examined proteins by immunohistochemistry are shown.

The median reading depth and percentage of covered target regions by NGS were 404 (97-586) and 92.75% (91.97%-94.61%), respectively. NGS detected a total of 72 types of point mutations in 26 genes, 19 deletions in 10 genes, and 3 insertions in 3 genes. Similar to the results of previous studies, variants of *TP53* and *RB1* were frequently found. As therapeutically targetable variants, mutations in *EGFR* (L858R), *KRAS* (G12D, G12A, G12V), and *PIK3CA* (E545K) were detected in 5 cases. The *PIK3CA* mutation (E545K) was not detected by NGS in 1 case (case 28), but was found by droplet digital polymerase chain reaction (ddPCR). One case (case 22) harbored both G12V and E545K mutations (Figure 1).

**Figure 2:**
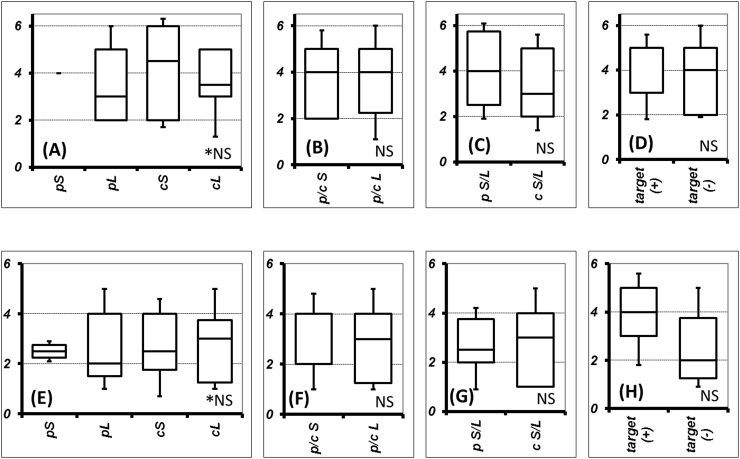
Comparison of genetic variant number concerning histological and/or component type or therapeutic-target The frequency of all genetic variants was compared among pure small cell lung cancer (SCLC), pure large cell neuroendrocrine carcinoma (LCNEC), combined SCLC, and combined LCNEC **(A)**. Frequency of genetic variants between p/c SCLC and p/c LCNEC **(B)**, pure S/L and combined S/L **(C)**, and cases with and without therapeutic targets **(D)**. The frequency of missense mutations was also compared among pure SCLC, combined SCLC, pure LCNEC, and combined LCNEC **(E)**. Frequency of missense mutations between p/c SCLC and p/c LCNEC **(F)**, pure S/L and combined S/L **(G)**, and cases with and without therapeutic targets **(H)**. Data are shown as the boundaries of the 10th, 25th, 50th, 75th, and 90th percentiles. Abbreviations: p, pure; c, combined; S, small cell lung cancer; L, large cell neuroendocrine carcinoma; ^*^NS, no significance between any subtypes; NS, no significance.

**Figure 3:**
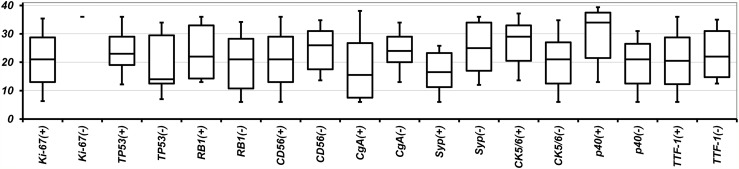
Number of genetic variants according to immunohistochemical profile Data are shown as the boundaries of the 10th, 25th, 50th, 75th, and 90th percentiles. Abbreviations: CgA, chromogranin A; Syp, synaptophysin.

**Figure 4:**
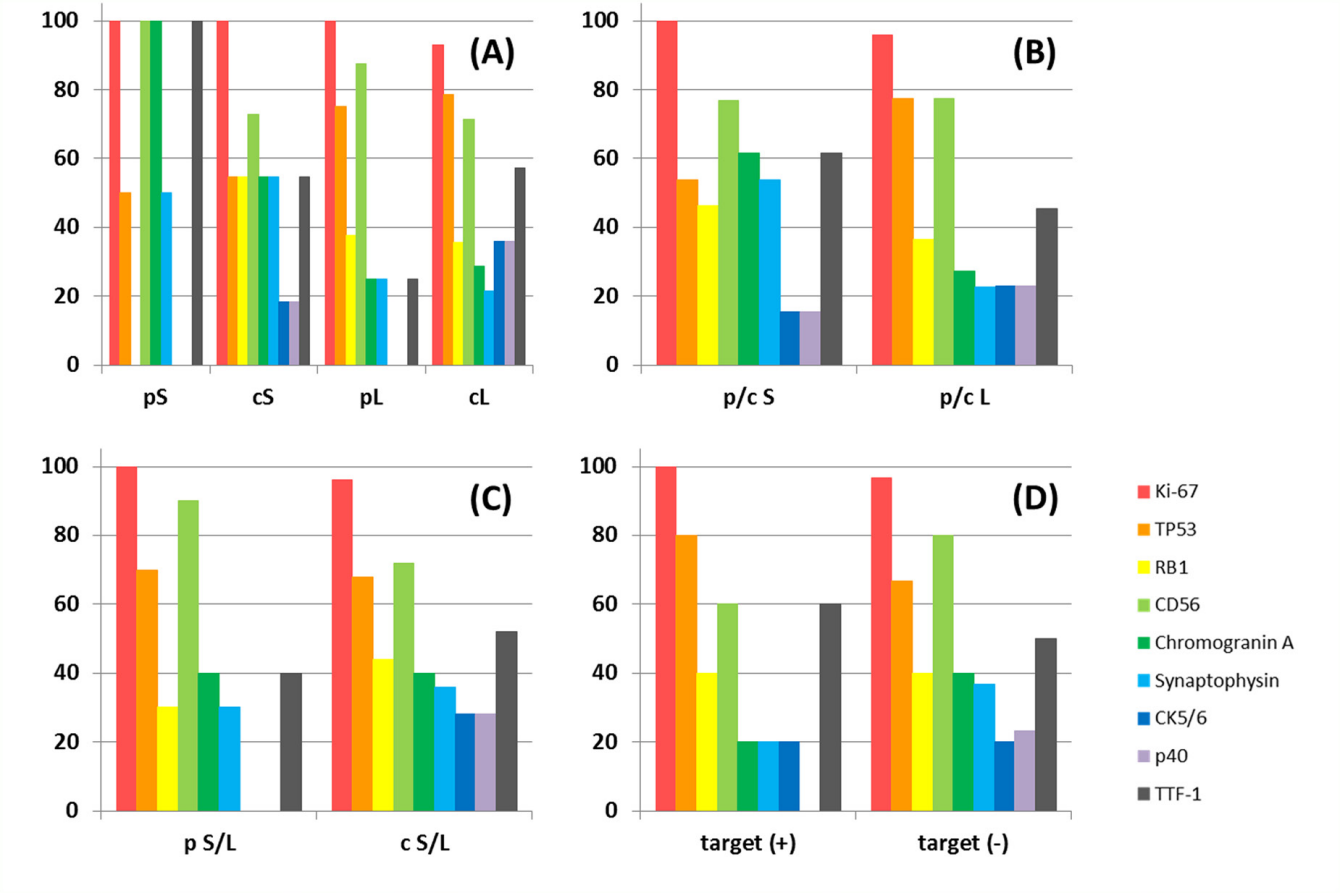
Percentage of positive cases by immunohistochemistry grouped by histological and/or component type or therapeutic-target The ratio of positive staining of the indicated antibodies was compared among pure small cell lung cancer (SCLC), combined SCLC, pure large cell neuroendocrine carcinoma (LCNEC), and combined LCNEC **(A)**. The ratio of positive staining between p/c SCLC and p/c LCNEC **(B)**, pure S/L and combined S/L **(C)**, and cases with and without therapeutic targets **(D)**. For each antibody, there was no significant difference in positive frequency in any of the comparisons. Abbreviations: p, pure; c, combined; S, small cell lung cancer; L, large cell neuroendocrine carcinoma.

According to the histological difference (SCLC or LCNEC) or intra-histological heterogeneity (pure type or combined type), cases can be divided into 4 subgroups. However, the frequency of genetic variants was not difference between any subtypes. There was also no statistically significant difference in the frequency of any genetic variation between SCLC and LCNEC, including when comparing the pure and combined types and cases with and without targetable variants. The statistical non-significance of the comparisons of SCLC and LCNEC did not change when the analysis was limited to missense mutations, which was the most common type of variant (Figure 2). Immunohistochemical status did not differ significantly in frequencies of genetic variants (Figure 3). Immunohistochemical profiling also did not show distinguishing features between SCLC and LCNEC, including when comparing the pure and combined types and cases with and without targetable variants (Figure 4).

**Figure 5:**
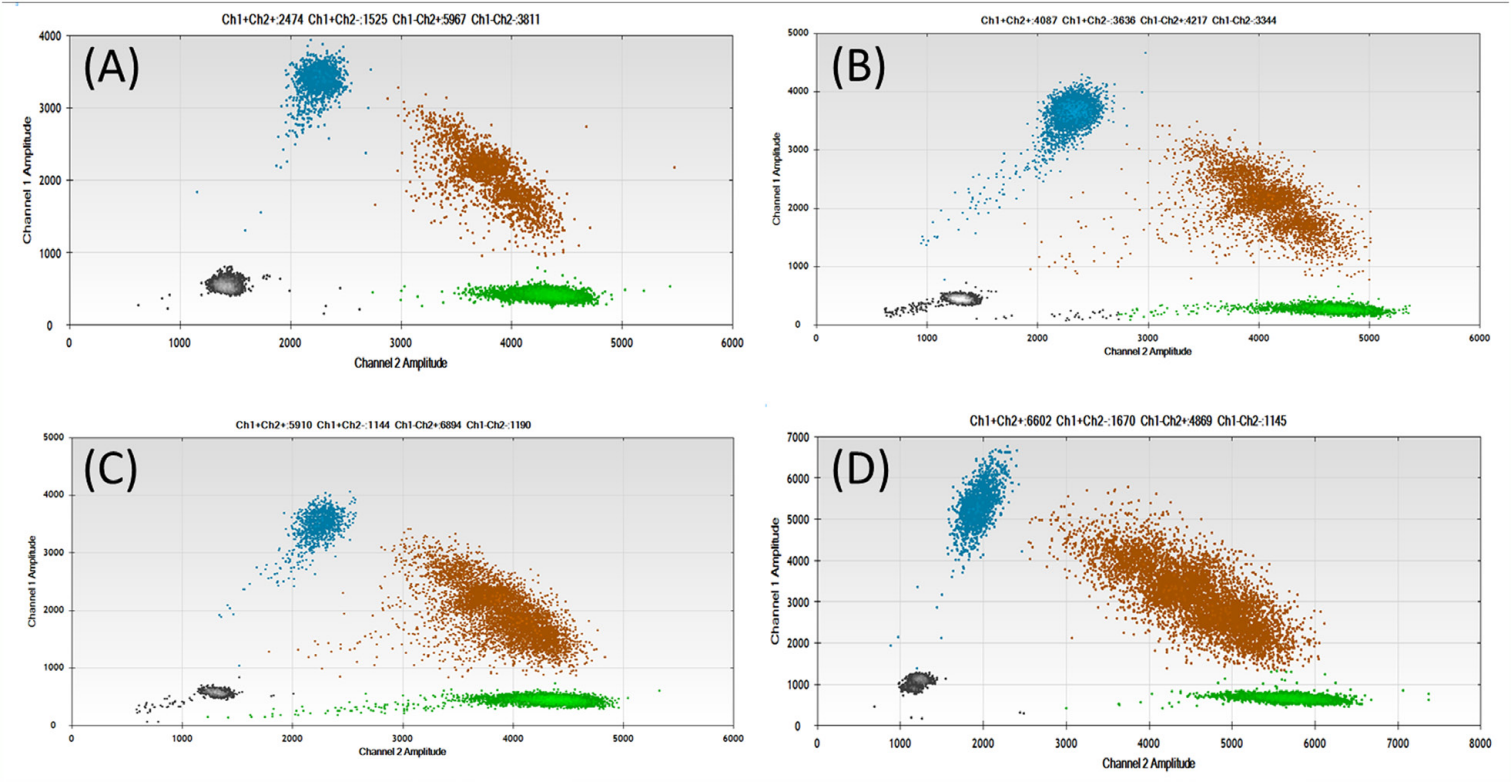
Results of droplet digital polymerase chain reaction of each variant Each amplified mutation of *KRAS* G12D **(A)**, G12A **(B)**, G12V **(C)**, and *PIK3CA* E545K **(D)** was visualized as a blue dot.

Apart from the L858R mutation, which was confirmed by an external examining body during a patient’s clinical course, therapeutically targetable variants detected by NGS were validated by ddPCR (Figure 5). The *KRAS* mutations G12D, G12A, and G12V were confirmed by the ddPCR *KRAS* screening multiplex kit (186-3506, BIO-RAD). The E545K mutation was confirmed by ddPCR with locked nucleic acid probes. Polymerase chain reaction (PCR) conditions and the sequence of each primer and probe are shown in Supplementary Table 1.

### Response to EGFR-tyrosine kinase inhibitors in a patient with combined LCNEC harboring the L858R mutation

**Figure 6:**
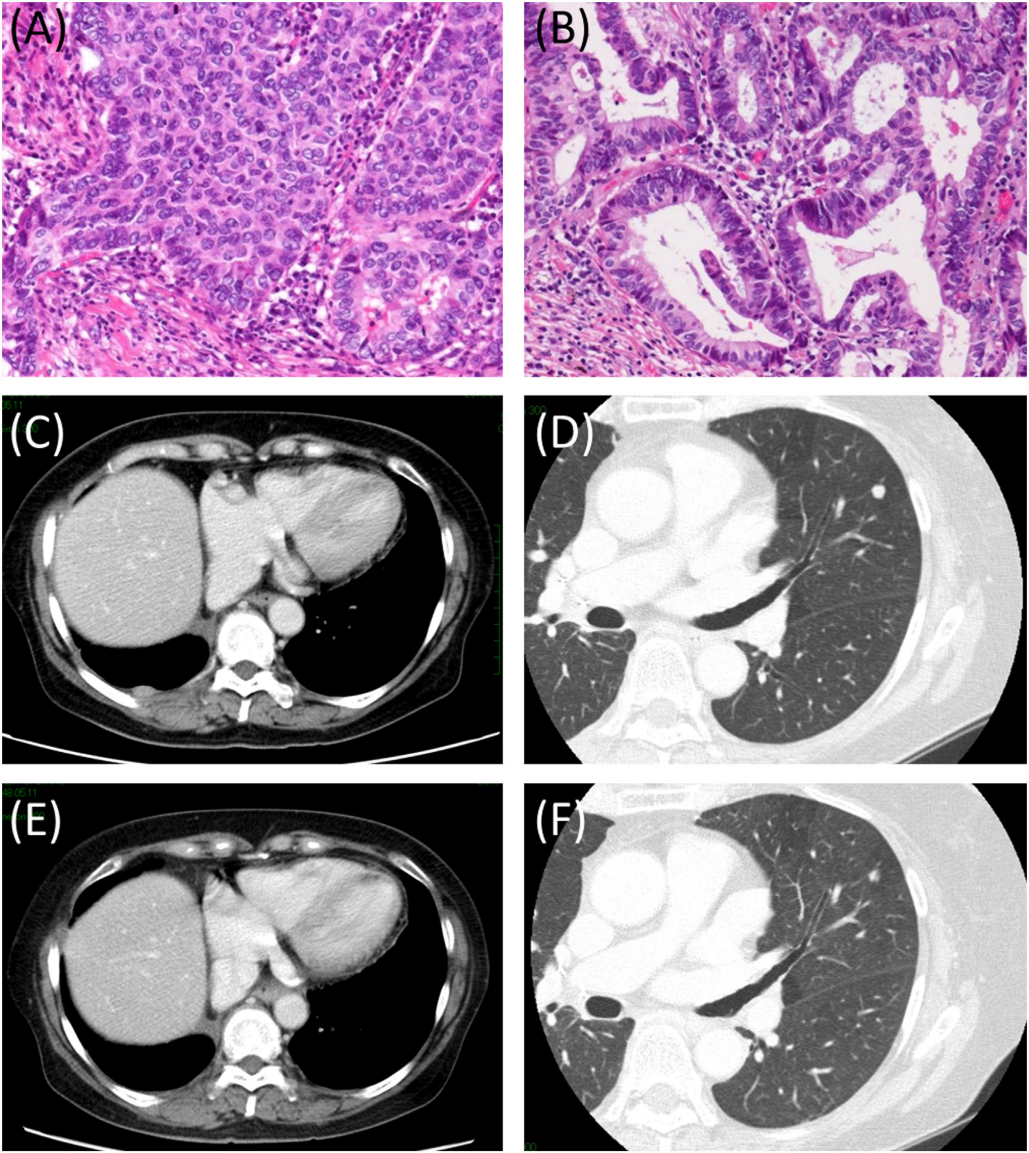
Histopathological findings and representative images of pre- and post-EGFR-TKI therapy A resected tumor was demonstrated to be combined LCNEC consisting of an LCNEC component **(A)** and an acinar component **(B)**. Ten months after resection, recurrences were detected at the right pleura **(C)** and left lung **(D)**. Both recurrence sites showed response to EGFR-TKI (E: right pleura, F: left lung). Abbreviations: EGFR-TKI, EGFR tyrosine kinase inhibitor; LCNEC, large cell neuroendocrine carcinoma.

The therapeutic response for tyrosine kinase inhibitor (TKI) was confirmed in 1 case that harbored the *EGFR* mutation (L858R). The patient was a 68-year-old woman who underwent screening, and pulmonary nodules and swelling mediastinal lymph nodes were detected. The tumor was diagnosed as a primary lung adenocarcinoma with mediastinal lymph node metastasis by biopsy. The patient was referred to the Hiroshima University Hospital and underwent chemotherapy with cisplatin/pemetrexed/bevacizumab. The tumor showed response to chemotherapy and was then resected surgically. Pathological examination demonstrated that the tumor was a combined LCNEC, consisting of an LCNEC component and an adenocarcinoma component. Ten months after resection, intrapulmonary and pleural recurrences were detected. After the L858R mutation was detected from the surgical specimen, the patient received EGFR-TKI and both metastases showed a response (Figure 6).

## DISCUSSION

SCLC and LCNEC of the lung are classified as subtypes of neuroendocrine tumor in the 2015 World Health Organization classification [1]. They are morphologically different, but have similar epidemiological features: for both, the typical patient is male and a smoker. Both SCLC and LCNEC often include components of other types of lung cancers, which are identified as combined SCLC or combined LCNEC. However, the clinical benefit of dividing the pure type from the combined type is not obvious. NGS studies have endeavored to reveal distinct genetic profiles of SCLC, LCNEC, adenocarcinoma, and squamous cell carcinoma of the lung [6–9]. In adenocarcinoma and squamous cell carcinoma, the existence of some genetic variants that could be targets of molecular therapy have been comprehensively analyzed [7, 8]. Studies from Western areas have not reported targetable genetic variations in SCLC or LCNEC [6, 9], whereas literature from Asia has indicated the existence of such targetable gene mutations [10, 11].

As a therapeutic target, mutations in *EGFR* are major genetic variants in adenocarcinoma, and it is known that adenocarcinomas with *EGFR* mutations can transform to SCLC after EGFR-TKI treatment [12–14]. Transformed SCLC is resistant to EGFR-TKI therapy. However, SCLC can also revert to adenocarcinoma after chemotherapy for SCLC and regain sensitivity to EGFR-TKI [12]. In regard to untransformed SCLC or LCNEC, the frequencies or clinicopathological features of cases harboring targetable mutations, including in *EGFR*, have not been adequately assayed by NGS.

Typical lung adenocarcinoma stems from alveolar type II cells or Clara cells. On the other hand, SCLC arises from neuroendocrine cells. Sutherland et al. indicated that alveolar type II cells can also give rise to SCLC [15]. Generally, adenocarcinoma is more likely to harbor targetable mutations compared to other types of lung cancer. If SCLC can stem from alveolar type II cells or neuroendocrine cells, the former might be more similar to adenocarcinoma in its clinical or genetic character. We hypothesized that if SCLCs or LCNECs harbor targetable mutations, then they stem from alveolar type II cells and are accompanied by adenocarcinoma-like features (i.e., are genetically similar to adenocarcinoma, histologically including an adenocarcinoma component, or positive for TTF-1 by immunohistochemistry). In the present study, the presence of targetable mutations in SCLC and LCNEC was assessed using NGS, and the cellular background and clinical features were evaluated by immunohistochemistry and genetic profiling to detect the specific features of cases harboring targetable mutations.

Among 35 cases, targetable mutations were detected in 5 cases. Several studies have indicated a therapeutic benefit of EGFR-TKIs in adenocarcinomas harboring an *EGFR* mutation [16, 17]. *KRAS* mutations and *PIK3CA* mutations, including E545K, are promising candidates for targeted therapy [18–24]. Some *BRAF* mutations or *ERBB2* insertions have also been reported as promising variants for targeted therapy [25–27]. However, the detected mutations in *BRAF* (G198C) and *ERBB2* (R113Q, R128Q, R143Q, S1020L, S1035L, S1050L) in our study were different from those previously reported. The clinical importance of these detected *BRAF* or *ERBB2* mutations was indeterminant.

A case with L858R (case 26) showed therapeutic response to EGFR-TKI therapy. Although *EGFR* mutation was confirmed from a surgical specimen and neither biopsy nor resection of recurrent sites was performed, we captured the LCNEC component and the adenocarcinoma component independently by laser microdissection and confirmed *EGFR* mutation in both components. Even when the tumor is of the combined type, the LCNEC component is representative of the highly malignant character of this tumor. Sequist et al. reviewed 79 cases and found no evidence of cytotoxic chemotherapy-induced transformation from adenocarcinoma to SCLC [16]. Considering that our patient did not receive TKI therapy until recurrence, it is likely that LCNEC component existed before cytotoxic chemotherapy and the tumor did not transform from adenocarcinoma to LCNEC, and recurrent sites were inferred to include the LCNEC component which showed response to EGFR-TKI.

Two NGS studies reporting *EGFR* mutations in SCLC and LCNEC did not evaluate the therapeutic sensitivity to EGFR-TKIs [10, 11]. The present study showed that NGS was useful in detecting targetable mutations in SCLC or LCNEC, and targeted therapy was valid in that case.

No significant difference was found between SCLC and LCNEC in terms of their genetic or clinicopathological profiles. Predictive features for cases harboring targetable mutations were also not detected. Rekhtman et al. suggested that LCNEC can be divided into non-SCLC-type and SCLC-type based on the frequency of *STK11*, *KEAP1*, or *NOTCH1-4* mutations [9]. This methodology was not valid in our study. Differences in race or study design with or without adenocarcinoma samples might account for this difference. It might be reasonable to place SCLC and LCNEC in the same category from the viewpoint of genetic mutations or possibility for targeted therapy. However, differentiating the pure type from the combined type may be less valid.

The major limitation of this study is the small number of cases. NGS studies including more SCLC/LCNEC cases, normal tissues as a control cohort, and tissues of other histological types of lung cancer as a comparison cohort are warranted. However, collecting a sufficient number of cases or adequate samples is not easy in SCLC/LCNEC of the lung.

In this study, the clinical benefit of targeted therapy was confirmed in patients harboring therapeutic targets that could be detected by NGS. In adenocarcinoma, the usefulness of NGS alone has been suggested for deciding the indication of targeted therapy [28]. In SCLC and LCNEC, a comprehensive evaluation like NGS should be performed not to miss the probability of target therapy.

## MATERIALS AND METHODS

### Study design

This study was approved by the Institutional Review Board (E-247) of Hiroshima University (Hiroshima, Japan). SCLC or LCNEC cases resected between January 2008 and March 2016 were retrospectively reviewed. Patients with available frozen sections were enrolled for NGS analysis, and clinicopathological data were collected from medical records. Frozen sections and formalin-fixed paraffin embedded tissues were utilized for NGS and immunohistochemical analysis, respectively. Therapeutically targetable mutations detected by NGS were validated by ddPCR.

### Next-generation sequencing

DNA was extracted from frozen sections using the QIAamp DNA Micro Kit (QIAGEN GmbH, Hilden, Germany). Extracted DNA was prepared for NGS using HaloPlex HS (Agilent Technologies, Santa Clara, USA) according to the manufacturer’s instructions and NGS was performed by MiSeq (Illumina Inc., San Diego, USA). Sequence reads were processed and mapped to a human genome reference sequence (hg19) using SureCall software 3.5.1.46 (Agilent Technologies, CA, USA). Genes whose variations could be therapeutically targetable or potentially useful for profiling tumor histological type were evaluated. The evaluated genes, number of targeted exons, and percentage of coverage for targeted regions are shown in Supplementary Table 2. Our custom panel was designed to cover 36 genes with a median coverage percentage of 99.57% (80.89–100).

Somatic gene variants were detected using Strand NGS software, Version 2.7, Build 229207 (Strand Life Sciences, Bangalore, India). After excluding variants that were neutral in the Mutation Assessor algorithm, damaging or deleterious mutations in other algorithms were regarded as significant. For the detection of deletions and insertions, after excluding neutral variants in the PROVEAN PREDICTION algorithm with a cutoff of -2.5, variants that were damaging or disease in the SIFT-indel or DDIG-in algorithms were determined to be significant.

Variants were defined as targetable if drugs have already clinically available (EGFR-TKIs) or there are persuasive enough references to consider them promising variants. For the latter, if variant was recognized as cancer-related in COSMIC and studies suggesting the variant as a promising target were identified in PubMed (literature was searched using the term of both “gene name” and “variant name”), the variants were picked up as targetable variants.

### Immunohistochemistry

Immunohistochemistry was performed with formalin-fixed paraffin embedded tissue samples. The antibodies used were as follows: Ki-67 (790-4286, Roche), p53 (ab80644, Abcam), RB1 (RB, SANTA CRUZ), CD56 (418191, NICHIREI), chromogranin A (412751, NICHIREI), synaptophysin (SYNAP-299-L-CE, Leica), cytokeratin 5/6 (M 7237, Dako), p40 (APR 3030A, BIOCARE MEDICAL), and TTF-1 (NCL-L-TTF-1, Leica).

### Droplet digital polymerase chain reaction

Somatic mutations that were potential therapeutic targets were validated by ddPCR using a QX100 Droplet Digital PCR (BIO-RAD, Hercules, USA). We designed primers and probes for the detection of PIK3CA and utilized the examination kit for the detection of KRAS.

### Statistics

The Mann-Whitney U test was used to evaluate statistical significance for numbers of genetic variants. For frequencies, statistical significance was evaluated using the Chi-square, Yates, or Fisher’s exact probability test. Probability values were derived from two-tailed tests and < 0.01 was considered statistically significant.

## SUPPLEMENTARY MATERIALS TABLES



## Abbreviations

ddPCR: droplet digital polymerase chain reaction
LCNEC: large cell neuroendocrine carcinoma
NGS: next-generation sequencing
PCR: polymerase chain reaction
SCLC: small cell lung cancer
TKI: tyrosine kinase inhibitor
