# Supplementary material for: Therapeutic strategies and genetic profile comparisons in small cell carcinoma and large cell neuroendocrine carcinoma of the lung using next-generation sequencing
Source: Oncotarget. 2017 Nov 14;8(65):108936–45. doi: 10.18632/oncotarget.22426 (PMC5752493; doi:10.18632/oncotarget.22426)
Supplement: Supplementary file 1 [file oncotarget-08-108936-s001.pdf]

Therapeutic strategies and genetic profile comparisons in small cell carcinoma and large cell neuroendocrine carcinoma of the lung using next-generation sequencing

SUPPLEMENTARY MATERIALS

Supplementary Table 1: Polymerase chain reaction (PCR) conditions and oligonucleotide sequences of primers and probes

E545K mutation	Oligonucleotide sequence (5′ → 3′)[b]
Primer[a]	
Forward	gacaaagaacagctcaaagcaa
Reverse	gcacttacctgtgactccatag
Locked nucleic acid probe[a]	
Wild type	caCTGAgCagg
Mutant type	tcaCTAAgCaGg

[a] Final concentrations of PCR primers, total and mutation probes, were adjusted to 900 and 250 nmol/L, respectively.
[b] Locked nucleic acid nucleotides are represented by uppercase letters and DNA nucleotides are represented by lowercase letters.

PCR conditions	
Initial denaturation at 95°C (10 min)	
Repetition of 40 cycles	94°C (30 s)<br>59°C (60 s)
One cycle at 98°C (10 min)	

Supplementary Table 2: Number of estimated exons and coverage percentage of estimated genes by HaloPlex

Gene	Number of estimated exons	Coverage (%)
AKT1	14	99.55
AKT2	17	99.44
AKT3	14	100
BRAF	21	96.36
CREBBP	31	100
EGFR	31	99.66
EP300	31	98.82
ERBB2	29	96.35
FGFR1	21	98.52
FMN2	20	80.89
IGF1R	21	100
KEAP1	6	100
KIAA1211	9	99.49
KIT	21	99.58
KRAS	6	96.74
MTOR	57	100
MYCL	3	100
NF1	63	97.99
NKX2-1	4	100
NOTCH1	34	99.87
NOTCH2	35	90.46
NOTCH3	33	99.87
NOTCH4	30	97.29
NTRK2	20	100
NTRK3	23	100
PIK3CA	20	87.81
PTEN	9	90.74
RB1	27	96.48
RBM10	24	100
SMARCA4	36	99.93
SOX2	1	100
STK11	11	100
TP53	14	90.65
TP73	14	100
TSC1	21	98.65
TSC2	43	97.98
